# Immunohistochemical Expression of Aquaporin-1 in Fluoro-Edenite-Induced Malignant Mesothelioma: A Preliminary Report

**DOI:** 10.3390/ijms19030685

**Published:** 2018-02-28

**Authors:** Giuseppe Angelico, Rosario Caltabiano, Carla Loreto, Antonio Ieni, Giovanni Tuccari, Caterina Ledda, Venerando Rapisarda

**Affiliations:** 1 Anatomic Pathology, Department of Human Pathology in Adult and Developmental Age “Gaetano Barresi”, University of Messina, 98122 Messina, Italy; giuangel86@hotmail.it (G.A.); aieni@unime.it (A.I.); tuccari@unime.it (G.T.); 2 Anatomic Pathology, Department of Medical and Surgical Sciences and Advanced Technologies, G.F. Ingrassia, University of Catania, 95124 Catania, Italy; rosario.caltabiano@unict.it; 3 Anatomy and Histology, Department of Biomedical Sciences and Biotechnologies, University of Catania, 95124 Catania, Italy; carla.loreto@unict.it; 4 Occupational Medicine, Department of Clinical and Experimental Medicine, University of Catania, 95124 Catania, Italy; vrapisarda@unict.it

**Keywords:** fluoro-edenite, Biancavilla, malignant pleural mesothelioma, cancer, asbestos, asbestiform, lung, NOA, biomarker

## Abstract

Background: The immunohistochemical expression of aquaporin-1 (AQP1) in asbestos-related malignant pleural mesothelioma (MPM) is emerging as a useful prognostic indicator of improved survival. A significantly increased incidence of MPM in a small town in southern Italy was ascribed to exposure to fluoro-edenite (FE), a naturally occurring asbestos fiber. We investigated the immunohistochemical expression of AQP1 in patients affected by FE-related MPM; taking into consideration its suggested independent prognostic role, its possible correlation with clinicopathological parameters and patient outcome was also evaluated. Methods: Ten patients were selected for this study, as neoplastic tissue blocks, clinical and follow-up data were available. The immunohistochemical overexpression of AQP1 was defined as ≥50% of tumor cells showing membranous staining. Results: Six cases showed AQP1 expression in ≥50% of tumor cells; in this group, a significant association of AQP1 overexpression with an increased median overall survival (OS) of 26.3 months was observed. By contrast, four patients exhibited an AQP1 score of <50% of stained cells, with a shorter median OS of 8.9 months. Conclusions: The present study represents further confirmation of the hypothesized prognostic role of AQP1, which seems a reliable prognostic indicator.

## 1. Introduction

Malignant pleural mesothelioma (MPM) is a highly aggressive neoplasm of the serosal membranes lining the pleural cavity and has been linked with exposure to asbestos fibers [1].

MPM results in a very poor prognosis because of a limited response to standard treatments, and the median survival is approximately 6 to 12 months [1,2]. Many diagnostic biomarkers have been proposed for MPM, such as calretinin, ck5, podoplanin, mesothelin, osteopontin, hyaluronic acid, fibulin-3 and vascular endothelial growth factor [3], but unfortunately no reliable prognostic predictors are currently clinically applied. 

The most important prognostic parameters for MPM include the histological subtype, sex and age at diagnosis [4]. Recent studies have highlighted the potential role of aquaporin-1 (AQP1) as an independent prognostic factor for improved survival in MPM patients exposed to asbestos [5,6]. AQP1 is a water channel protein found in cell membranes throughout the body and facilitates transcellular water transport [7,8]. In MPM cases, AQP1 has a role in cell proliferation, adhesion and motility, as well as in the modulation of pleural fluid volumes [9,10,11,12]. Furthermore, an increased expression of AQP1 evaluated by immunohistochemistry in asbestos-related MPM has been documented as a possible predictor of better prognosis. In detail, according to two recent studies on large series, high levels of immunohistochemically expressed AQP1, in over 50% of tumor cells, seemed to be linked with improved overall survival (OS) in these patients [5,6]. 

The majority of MPM cases are associated with exposure to asbestos fibers; however, other asbestos-like fibers have been considered agents to promoting MPM, such as erionite and fluoro-edenite (FE) fibers. [13,14,15,16,17,18,19,20,21,22,23]. The latter kind of fiber has raised the attention of the scientific community as a result of an increase in the incidence and mortality of MPM in Biancavilla, a small town on the southwest slope of Mt. Etna in Sicily (southern Italy), as revealed by an epidemiological survey conducted from 1988 to 1997 [24,25]. Environmental investigations showed the presence of FE fibers in the lava rocks excavated from a local stone quarry and used locally for about 50 years for house and road construction building purposes. In vitro, in vivo and epidemiological studies have demonstrated that FE fibers share size and morphological similarities with tremolite amphibolic asbestos fibers [13,14,15,16,17,18,19,20,21,22,23,24,25,26,27]. Therefore, FE fibers have been declared carcinogenic to humans by the International Agency for Research on Cancer (IARC; Lyon, France) [28].

In the present study, we investigated the immunohistochemical expression of AQP1 in a small but unique subset of MPM patients living in the Biancavilla area with documented evidence of environmental exposure to FE fibers. We also evaluated the correlation between AQP1 immunohistochemical expression and clinico-pathological parameters.

## 2. Results

The cohort of MPM FE-related patients was composed of six men and four women (mean age: 68.4 years; age range: 50–93 years). According to the World Health Organization (WHO) criteria, six cases were histologically classified as epithelioid, one was classified as sarcomatoid, and three were classified as biphasic subtypes [29]. In detail, two biphasic MPM cases from our series showed a slight predominance of the sarcomatoid elements (60% sarcomatoid, 40% epithelioid), while one case consisted predominantly of sarcomatous elements with scattered glandular elements. All clinico-pathological and immunohistochemical data are summarized in Table 1. 

**Figure 1:**
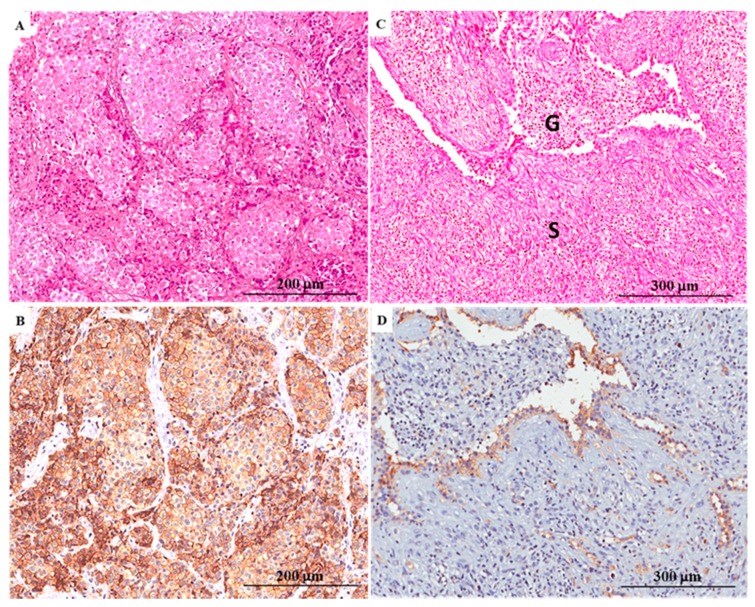
Histology and aquaporin 1 (AQP1) immuno-expression in malignant pleural mesothelioma (MPM) cases. (**A**) Haematoxylin and eosin (H&E) stained section demonstrating a malignant neoplasm with a solid pattern of growth composed of epithelioid cells (×200); (**B**) The majority of neoplastic cells exhibited a positive immunohistochemical stain for AQP1. In detail, a linear and circumferential membranous staining with strong intensity was recorded (×200); (**C**) H&E stained section of a case of biphasic mesothelioma composed predominantly of neoplastic sarcomatoid cells with a spindled morphology (S) and occasional glandular structures (G) (×100); (**D**) AQP1 immunostain was recorded in <50% of tumor cells and was localized exclusively in the tumor areas with epithelial differentiation (×100).

**Figure 2:**
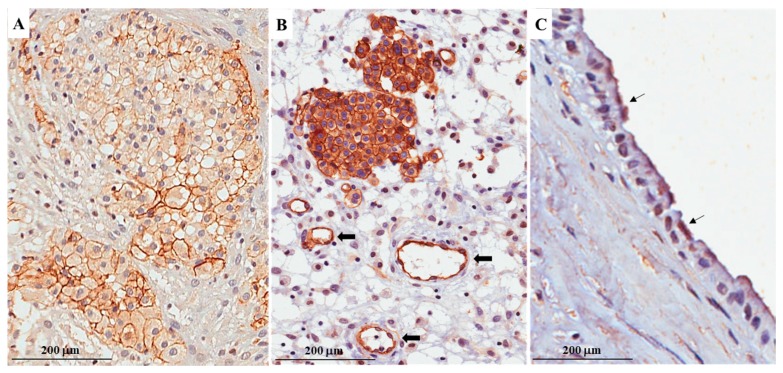
(**A**) Linear and circumferential membranous staining for aquaporin-1 (AQP1) is depicted; (**B**) Vascular endothelial cells were used as positive control for AQP1 immunostain (arrows); (**C**) For comparison, the AQP1 immunohistochemical stain on non-neoplastic mesothelium is shown; in detail, the immunostain was exclusively localized to the apical cellular portion of mesothelial cells (arrows).

On parallel sections obtained from neoplastic tissue blocks, the immunohistochemical expression of AQP1 was documented by the linear (partial) and circumferential (complete) membranous staining not exclusively lining the apical cellular portion (Figure 1 and Figure 2). 

**Figure 3:**
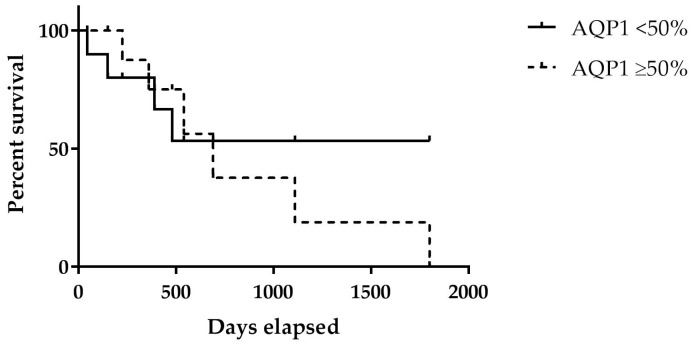
Kaplan-Meier survival curve of aquaporin-1 (AQP1) expression in fluoro-edenite (FE)-induced mesothelioma patients.

Taking into consideration that only a score of ≥50% was considered as overexpression, six (60%) cases showed this AQP1 pattern; in this group, the Kaplan–Meier method showed a significant association of AQP1 overexpression with an increased OS, and the hazard ratio was 1.492 with a 95% confidence interval (CI) (0.432–5.153) (Figure 3; Table 2).

This difference in the median OS between high and low AQP1 expressors appeared statistically significant (*p* < 0.05).

Moreover, all MPM cases showed positive immunostains for calretinin, cytokeratin 5/6 and Wilms tumor protein (WT1), thus confirming the mesothelial histogenesis of FE-related MPM; by contrast, no immunoexpression was recorded for cytokeratin 7 or thyroid transcription factor (TTF1), consequently excluding a possible pulmonary origin of neoplasms. 

No relationship emerged between AQP1 expression and other clinico-pathological variables. 

## 3. Discussion

In the present study, we started a systematic analysis of a cohort of MPM patients living near the Biancavilla area, where the increased incidence and mortality from MPM have been attributed not to traditional asbestos fibers but to FE fibers.

In particular, we performed an immunohistochemical investigation on the AQP1 expression and distribution in MPM, also taking into consideration the suggested prognostic significance of AQP1 in asbestos-related MPM samples, as previously reported elsewhere [5,6,30]. 

In detail, AQP1 overexpression was revealed in 60% (*n* = 6) of MPM FE-induced cases, while the other 40% (*n* = 4) of cases exhibited a negative AQP1 expression or sometimes focal/non-uniform staining in less than 50% of the neoplastic mesothelial elements. This different AQP1 immunoreactivity appeared to be able to greatly influence the final outcome for MPM FE-induced patients; in fact, a significantly longer OS was found in the group with AQP1 overexpression, with delayed recurrences and death for the disease. By contrast, earlier recurrences and the worst prognoses were encountered in four patients who showed a low immunohistochemical expression of AQP1. In detail, a statistically significant association of AQP1 overexpression with increased survival was observed with a mean OS of 26.3 months for patients with ≥50% AQP1 expression versus a mean OS of only 8.9 months for patients with <50% AQP1 expression. This relationship between higher levels of AQP1 in MPM tissues and a better prognosis is quite surprising, as it appears to be in contrast to that reported in other tumors, including breast cancer, brain tumors, prostate adenocarcinoma, lung adenocarcinoma and carcinomas of the gastrointestinal tract, for which increased levels of AQP1 are associated with a poorer prognosis [12,31,32,33,34,35,36,37,38]. However, the role of AQP1 overexpression as an independent prognostic factor of a better prognosis has already been documented in MPM patients exposed to asbestos. Moreover, similar results to those observed for MPM have also been documented in receptor tyrosine-protein kinase erbB-2 (HER2)-positive early breast cancer, in patients with colorectal cancer and in biliary tract carcinoma, where AQP1 overexpression detected by immunohistochemistry was significantly associated with improved survival [39,40,41]. In the present study, AQP1 overexpression appeared as a promising prognostic tool for MPM patients; however, the relatively small number of MPM FE-induced patients followed indicates further validation is required to establish the definite prognostic value of AQP1 overexpression. Moreover, the principal limitation of the present study was the impossibility to retrieve the clinical stage for all studied patients. Therefore, our future perspective is to demonstrate the role of AQP1 as an independent prognostic marker also when it is corrected for disease stage.

We believe that in future studies, the clinical and prognostic implications of AQP1 expression should be integrated with other prognostic and predictive parameters. In this regard, the tumor immune microenvironment and the complex interrelations between tumor cells, mesenchymal stem cells and cells of the immune system have recently been found to play an important role in MPM tumorigenesis [42,43].

## 4. Materials and Methods

### 4.1. Sample Collection

Although the present research complied with the Helsinki Declaration, the non-interventional retrospective nature of our study did not require any informed consent by the local research ethics committee. 

Adequate bioptic tissue from 10 patients who underwent video-assisted thoracoscopic surgery for MPM between 1996 and 2014 were retrospectively obtained from a larger series of 49 patients, for whom clinico-pathological reports and follow-up data were available.

The information for each patient came from the National Registry of Mesotheliomas (Renam). For each of the 10 analyzed subjects, the MPM diagnosis had been certified with cytological and histological exams. 

All patients were residents in the town of Biancavilla or in nearby areas. 

The histological diagnosis of MPM and histological subtypes were determined in accordance with the WHO criteria.

### 4.2. Laboratory Tests

Sections (4–5 μm in thickness) were cut from paraffin blocks using a microtome, mounted on sialinate-coated slides (Dako, Glostrup, Denmark) and stored at room temperature. The sections were stained with hematoxylin and eosin (H&E) and examined using a Zeiss Axioplan light microscope (Carl Zeiss, Oberkochen, Germany) for general morphological characterization and to highlight the presence or absence of structural alterations.

For diagnostic purposes, by immunohistochemistry, 4 μm sections of tissue samples from the same patients were stained using a Ventana Benchmark immunostainer (Ventana Medical Systems, Inc., Oro Valley, AZ, USA). In each case, a set of the following commercially obtained monoclonal antisera was applied: calretinin (SP65, Roche Diagnostics, Indianapolis, IN, USA; working dilution (wd) of 1:150), cytokeratin 7 (SP52, Roche Diagnostics; wd of 1:1000), cytokeratin 5/6 (D5/16B4, Roche Diagnostics; wd of 1:250), WT1 (6F-H2, Roche Diagnostics; wd of 1:250), and TTF1 (SP141, Roche Diagnostics; wd of 1:200). 

Moreover, on parallel sections, AQP1 (B-11, Santa Cruz Biotechnology, Santa Cruz, CA, USA; wd of 1:100) was applied. In addition, as elsewhere suggested, the percentage of immunostained cells was assessed by semi-quantitative optical analysis according to a four-tiered system (0 = negative; <25% positive cells = focal staining; ≥25 to <50% positive cells = not uniform staining; ≥50% positive cells = diffuse staining); cases with over 50% immunoreactive neoplastic cells were considered as evidence of AQP1 overexpression, as reported elsewhere [30]. 

For the quantitative evaluation, the percentage of cells labeled by the antibodies was blindly assessed by two qualified anatomic pathologists (R.C. and G.T.). Only membrane labeling was considered specific, and this pattern of labeling was confirmed from 10 high-power (×400) fields (Figure 2A). In the case of dispute concerning interpretation, the case was reconsidered by a double-headed microscope to reach agreement. Positive and negative controls for AQP1 were used to test the specific reaction of the primary antibody used in this study at the protein level. Vascular endothelial cells and non-neoplastic mesothelial cells served as positive internal controls (Figure 2B). Negative controls, involving the omission of the primary antibody, were included.

Finally, representative photomicrographs were captured using a digital camera (AxioCam MRc5, Carl Zeiss).

### 4.3. Statistical Analysis

The OS was the primary endpoint for this study and was calculated from the date of diagnosis of the MPM FE-related patients and the date of death. The hazard ratio was calculated using the Mantel-Haenszel test. Cancer-specific survival analysis was performed using the Kaplan-Meier method, and for comparison of the survival curves, the Mantel-Cox log-rank test was used. A univariate model was evaluated for AQP1 expression; age, sex and histologic subtype were entered in the multivariate model. A *p*-value of less than 0.05 was considered statistically significant. Statistical analysis was performed using GraphPad Prism version 7 (GraphPad Software, Inc., La Jolla, CA, USA).

## 5. Conclusions

We contend that, together with the well-known suggested immunohistochemical mesothelial markers, a promising role should be attributed to AQP1, its overexpression being identified as a promising better prognostic predictor reliable also in cytopathology [38].

In conclusion, our study represents the first report that emphasizes the immunohistochemical expression of APQ1 occurring in MPM cases related to environmental exposure to FE fibers. We will continue our efforts in the already identified larger cohort to definitively establish the APQ1 prognostic significance.

## Figures and Tables

**Table 1:** Clinicopathological features of all studied cases of fluoro-edenite (FE)-related malignant pleural mesothelioma (MPM).

Case	Age	Sex	Pathological Subtype	Survival Time (Months)	Aquaporin-1 Expression
1	69	M	Epithelioid	1.5	<50%
2	50	M	Biphasic 20% epithelioid, 80% sarcomatoid	16	<50%
3	69	F	Sarcomatoid	5	<50%
4	74	F	Epithelioid	13	<50%
5	85	M	Epithelioid	23	≥50%
6	93	F	Biphasic 40% epithelioid, 60% sarcomatoid	7.5	≥50%
7	58	F	Epithelioid	18	≥50%
8	55	M	Epithelioid	37	≥50%
9	75	M	Biphasic 40% epithelioid, 60% sarcomatoid	60	≥50%
10	56	M	Epithelioid	12	≥50%

**Table 2:** Univariate and multivariate analysis for aquaporin-1 (AQP1) expression.

	Univariate Analysis HR (95% CI)	*p*-Value	Multivariate Analysis HR (95% CI)	*p*-Value
<50%	1	<0.05	1	<0.05
≥50%	1.5 (0.43–5.15)		1.8 (0.85–4.72)	

HR = Hazard Ratio; CI = Confidence interval.

## Abbreviations

AQP1	Aquaporin-1
CI	Confidence interval
FE	Fluoro-edenite
MPM	Malignant pleural mesothelioma
H&E	Haematoxylin and eosin
WT1	Wilms tumor protein
TTF1	Thyroid transcription factor
OS	Overall survival
